# ANTS 2020 Special Issue: Editorial

**DOI:** 10.1007/s11721-021-00208-3

**Published:** 2021-11-23

**Authors:** Marco Dorigo, Thomas Stützle, Maria J. Blesa, Christian Blum, Heiko Hamann, Mary Katherine Heinrich

**Affiliations:** 1 IRIDIA, Université Libre de Bruxelles, Brussels, Belgium; 2 Universitat Politècnica de Catalunya, Barcelona, Spain; 3 Artificial Intelligence Research Institute, IIIA-CSIC, Barcelona, Spain; 4 University of Lübeck, Lübeck, Germany

This special issue of the *Swarm Intelligence* journal is dedicated to the publication of extended versions of some of the best papers presented at *ANTS 2020, Twelfth International Conference on Swarm Intelligence*, which took place in Barcelona, Spain, on October 26–28, 2020. Due to the COVID-19 pandemic, the conference was held online.

ANTS is the first and most established conference series dedicated to the dissemination of swarm intelligence research. Its first edition took place in 1998, at the Université Libre de Bruxelles, Brussels, Belgium. Since then, it has been held every other year and, since 2010 (i.e., starting with the seventh edition of the conference), the authors of full conference papers have been invited to submit an extended version for possible inclusion in a dedicated special issue of the *Swarm Intelligence* journal.

Five articles from the 2020 edition of the ANTS conference were accepted for publication in the special issue after at least two rounds of reviews with comments by at least three referees. The papers co-authored by a guest editor of this special issue have been managed anonymously by one of the other guest editors. In the following, each paper is briefly introduced by the guest editor who managed it.

The special issue opens with “*Robot swarm democracy: the importance of informed individuals against zealots*.” In this paper, Giulia De Masi, Judhi Prasetyo, Raina Zakir, Nikita Mankovskii, Eliseo Ferrante, and Elio Tuci study the interplay between three types of agents—zealots, informed, and uninformed—in the context of the well-known best-of-n collective decision-making problem. Their results show that when all robots are informed (i.e., capable of measuring the quality of the different options), the swarm converges to the best option even if the number of zealot agents steering the swarm toward low-quality options is higher than those steering the swarm toward the high-quality option, provided the unbalance of zealots is not too high. They also show that informed agents can be effectively used to counteract the negative effect of zealot agents that try to steer the swarm towards low-quality options. The study was performed using mathematical models and simulations, and the obtained results were confirmed with real robot experiments.

In “*HuGoS: A virtual environment for studying collective human behavior from a swarm intelligence perspective*,” Nicolas Coucke, Mary Katherine Heinrich, Axel Cleeremans, and Marco Dorigo present a multi-user virtual environment that supports the study of human interactions and group behaviors. Human interactions and group behaviors are often more complex and variated than those displayed by animals or artificial agents. Moreover, human relations have unique aspects of human psychology that are not usually studied in swarm intelligence research. The presented multi-user virtual environment named HuGoS—Humans Go Swarming—is an open-source software tool implemented using the Unity game development platform. HuGoS turns out to be a versatile tool that allows the implementation of a large number of possible scenarios, making it a comprehensive tool for experimentation. The authors show the functionality of HuGoS experimentally via a coordination task performed by anonymous human participants. The performance of the task includes group activities such as dynamic best-of-n collective decision making, messaging or signaling, and stigmergic interactions. HuGoS is a step forward into the research on human swarm intelligence, and it will hopefully help in acquiring a better understanding of the new and complex behaviors arising in larger human collectives.

In “*Discrete collective estimation in swarm robotics with distributed Bayesian belief sharing*,” Qihao Shan and Sanaz Mostaghim present a novel approach to collective decision making in robot swarms based on Bayesian hypothesis testing. While most of the previous work has focused on the study of simple methods with minimal requirements in terms of computational complexity and communication bandwidth, this novel approach explores the benefits of a more complex method that is a decentralized variant of a previously published approach that required a leader. As benchmarking scenario, the authors chose the well-known collective perception task where robots in a swarm estimate a globally distributed environmental feature: the ratio of black and white tiles covering the experimental floor. Individual robots rely on Bayesian statistics to assess the most likely state of the environment and exchange their beliefs by communicating with neighbors. The authors compare their approach to state-of-the-art methods, such as direct comparison and voter-based decision making. The results show that this new approach shows good accuracy and speed at the cost of more communication between the robots.

In “*Ant colony optimization for feasible scheduling of step-controlled smart grid generation*,” Jörg Bremer and Sebastian Lehnhoff describe the application of ant colony optimization to an interesting real-life problem: the scheduling of step-controlled devices in the management of energy resources. This problem, which is NP-hard from a mathematical perspective, will become more and more relevant in the near future due to the gradual shift in the electrical energy grid from a few large power plants to many rather small and decentralized energy producers. One of the main challenges for solving the problem considered in the paper is the construction of feasible solutions. For this purpose, the authors enhance the proposed ant colony optimization algorithm with a simulation model used for the identification of feasible paths during solution construction. The authors also present a study on the complexity and structure of the underlying feasibility landscape.

In “*Resource ephemerality influences effectiveness of altruistic behavior in collective foraging*,” Johannes Nauta, Yara Khaluf, and Pieter Simoens study the link between altruism and foraging efficiency. In the altruistic behavior they study, an agent prioritizes collective intake over individual intake–it will recruit other members of its species to feed on a resource patch instead of feeding itself. The authors show that the net gain of engaging in altruistic foraging behavior depends on the durations of impermanent resource patches. When a patch has a longer duration, the patch should induce altruistic behavior to increase foraging efficiency. Conversely, when a patch has a shorter duration, an agent should feed on it individually before it disappears. The authors show that the net gain also depends on the overall availability of resource patches. Altruistic behavior increases foraging efficiency when patches are sufficiently difficult to locate but not when patches are readily available. The authors also show that the effectiveness of altruistic behavior during foraging depends on the density and communication range of the agents. Altruistic behavior is more effective than other strategies when the incidence rate of agent-to-agent communication is low but not necessarily when the incidence rate is high.

The high quality of the five papers contained in this special issue is the result of the collaboration of a large number of people: the authors, who submitted their best work to the journal; the referees, who helped in the selection of the published papers; and finally, the many people at Springer who assisted us in the production phase. We thank them all for their help to make this special issue possible.

